# Editorial: Alignment options and robotics in total knee arthroplasty (TKA) “Alignment, alignment, alignment, but TKA fail mainly for infections and instability”

**DOI:** 10.3389/fsurg.2023.1247759

**Published:** 2023-07-11

**Authors:** Pier Francesco Indelli, Andrea Giordano Salvi, Giuseppe Petralia

**Affiliations:** ^1^ Division of Orthopaedic Surgery, Südtiroler Sanitätsbetrieb, Brixen, Italy; ^2^ Division of Orthopaedic Surgery, Institute for Biomedicine, EURAC Institute, Bozen, Italy; ^3^ Department of Orthopaedic Surgery, Stanford University School of Medicine, Redwood City, Stanford, CA, United States; ^4^ Personalized Arthroplasty Society (PAS), One Glenlake Parkway NE, Atlanta, GA, United States; ^5^ Dipartimento di Medicina Clinica, Sanita’ Pubblica, Scienze Della Vita e dell’Ambiente, Universita’ degli Studi dell’Aquila, L’Aquila, Italy

**Keywords:** TKA, alignment, kinematic—joint mobility analysis, total knee anthroplasty, robotics [MeSH], kinematic alignment, periprosthetic joint infection (PJI), instability

**Editorial on the Research Topic** Alignment options and robotics in total knee arthroplasty

Although adult reconstruction surgery represents an effective solution for symptomatic end stage knee osteoarthritis, only a limited percentage of patients ultimately meet their expectations after total knee arthroplasty (1). One of the reasons of these not always satisfactory outcomes, seems to be the anatomic changes in knee kinematic following the application of the principle of the classic mechanical alignment technique because this alignment philosophy is not able to restore the pre-arthritic bone morphotype of most of the knees (2, 3). Therefore, multiple authors from different countries recently challenged the dogma that mechanical alignment represents the gold standard for TKA: Howell et al. introduced (2) the “kinematic alignment” (KA), Vendittoli et al. (3) proposed the “restricted KA” (rKA), Winnock de Grave et al. the “inverse KA” (4) and Lustig et al. (5) the “functional alignment”.

Both classical kinematic alignment and restricted kinematic alignment techniques aim to restore the soft tissue laxity through pure resurfacing of femur, eventually changing the tibial coronal and sagittal slope to cope with eventual gap unbalance. Nevertheless, the restricted kinematic technique defines a “safe zone” for TKA alignment to avoid reproducing extreme pathoanatomies, due to a potential higher risk of aseptic loosening. On the other hand, the inverse KA rational is to resurface the tibia, maintaining the orientation of its joint line obliquity, because a tibial recut could potentially lead to a difficult to manage flexion-extension gap unbalance. Lasty, the functional alignment technique represents an evolution of the KA concept by deploying Computer Assisted Surgery (CAS) technologies to be more accurate in bone resections and components position. CAS represents a fast-evolving field, due to the need of a higher accuracy during the surgical workflow to restore the joint kinematic, and robotic surgery ultimately represents a modern evolution of CAS platforms.

In this research topic section of Frontiers in Surgery, Massé et al.' presented a robot assisted personalized surgical technique based on the recreation of a pre-arthritic anatomy within a range of acceptable alignment boundaries: this technique follows pure measured-resection principles with the goal of maintaining the native soft tissue tension as highlighted by Vendittoli et al. (3).

The Rosa Robotic System (Zimmer Biomet, Warsaw, Indiana) utilizes anteroposterior and lateral radiographs to create 3D bone models, representing a form of image-based robotic technology. The authors present their technique in a step-by-step modality, guiding the readers through the entire procedure. Both the Editors and the reviewers appreciated the quality of the manuscript, but two main issues are still pending.

Firstly, even if the proposed surgical technique can restore the pre-arthritic bone morphology of the knee, the restoration of the pre-arthritic whole anatomy, as well as the kinematic, cannot be guarantee. It is our opinion that the reason of this potential deficit is the lack of an accurate and reproducible soft tissue assessment during the procedure, as stated by the same authors. Moreover, in severe longstanding joint coronal deformity, the soft tissue envelop may already been disrupted and the re-constitution of the original ligament tension could be only hypothesized by the surgeon own experience, as shown by Valpiana et al. in a similar study presenting an “hybrid” robot-assisted restricted kinematic alignment surgical technique (6). For this reason, up to date, many modern forms of CAS are not able to prevent the dynamic instability that represent the second major cause of TKA failure (7).

Secondly, the time seems to have come to better define what sagittal alignment “safe range” really means: in fact, thanks to CAS data, surgeons can finally better understand the impact of the tibial slope and femoral flexion on the knee kinematic. This could be paramount namely in post-traumatic cases where a tibial or femoral abnormal bowing, not only in the coronal plane, but in the sagittal plane too, frequently exists. Knowing how much the articular bone resection will correct a sagittal lower limb deformity, without jeopardizing the functional outcome or increasing the risk of aseptic loosening, could be one of the main goals of any personalized TKA technique.

In the second article published in the current research topic, Wen et al., presented a retrospective study comparing, functionally and radiologically, two groups of patients who underwent kinematic alignment (KA) or mechanical alignment (MA) TKA. Besides to be the first study in mainland China to report comparative results between KA and MA with a 2-year minimum FU, the finding of this study is interesting because it highlights that CAS is not always mandatory to reach good results. In fact, in the KA-TKA group, a high level of satisfaction and a satisfactory functional outcome have been achieved utilizing traditional instrumentation: this finding keeps the debate on the spread use of CAS platforms still open. In this study, this finding was obviously the consequence of patient recruitment selection criteria since the authors decided to exclude extreme anatomies and valgus deformity. Ultimately, the “take home message” from this article is represented by the recommendation of using CAS only in cases where it represents a real game changer to obtain the ideal result. However, this report suggests that a meticulous pre-operative assessment of patients is mandatory to screen for a pathoanathomy which may ultimately be too challenging for a traditional KA instrumentation.

In the third and last article published in the current research topic, Neufeld et al. presented a pilot study to determine current practices, opinions, and understandings among orthopaedic surgeons in Canada regarding use of surgical tourniquets in TKA. The decision on the use of a tourniquet is still surgeon dependent and clear recommendations are still lacking: the authors underlined that, despite the bioengineered evolution of tourniquet systems and despite the current literature suggests that its proper use could minimize risks, only a fraction of Canadian surgeons is currently using this device routinely. Interestingly, the authors recommend that the tourniquet pressure should be based on Limb Occlusion Pressure (LOP) instead than empirically. The authors conclusion, anyway, confirms a lack of evidence, in the current literature, on the safety and efficacy of tourniquet use in TKA.

Unfortunately, this research topic leaves a question unsolved: does the addition of robotic technology in the operating room have a negative impact on orthopaedic surgery residents and adult reconstruction fellows surgical skills and understanding of fundamental concepts of TKA? A recent study showed that 70% of US trainees are having some degree of exposure to robotic assisted TKA during residency with many of them feeling to receive educational benefits to training with robotic assistance in the operating room but also a substantial portion feeling that robotic assistance was detrimental to their training (8).

In conclusion, the research topic Alignment Options and Robotics in TKA has been investigated mainly by two authors (VM and LW): both studies support the recent, growing evidence in the current literature that TKA patients may benefit from a form of personalized alignment. The use of computer assisted surgery is helping surgeons in an accurate reproduction of the surgical plan (preoperatively as well as intraoperatively) but the real benefit from the use of advanced technologies and modern surgical techniques in order to reduce the incidence of the two main causes for TKA failure (periprosthetic joint infections and instability during the gait cycle) still needs to be demonstrated. In 2023, static frontal limb alignment is still known to be a poor predictor of knee kinematics (9, 10).
